# The efficient generation of knockout microglia cells using a dual-sgRNA strategy by CRISPR/Cas9

**DOI:** 10.3389/fnmol.2022.1008827

**Published:** 2022-10-13

**Authors:** Mengfei Zhang, Fang Yi, Junjiao Wu, Yu Tang

**Affiliations:** ^1^Department of Geriatrics, Xiangya Hospital, Central South University, Changsha, China; ^2^National Clinical Research Center for Geriatric Disorders, Xiangya Hospital, Central South University, Changsha, China; ^3^Department of Rheumatology and Immunology, Xiangya Hospital, Central South University, Changsha, China; ^4^Provincial Clinical Research Center for Rheumatic and Immunologic Diseases, Xiangya Hospital, Central South University, Changsha, China

**Keywords:** LRRK2, microglia, Parkinson's disease, dual-sgRNA, CRISPR/Cas9

## Abstract

Gene deletion in microglia has become an important and exciting approach for studying neuroinflammation, especially after the development of the CRISPR/Cas9 system for genome editing during the last decade. In this study, we described a protocol for the highly efficient generation of knockout microglia cells using a dual-short guide RNA (sgRNA) strategy by CRISPR/Cas9. *Leucine-rich repeat kinase 2 (LRRK2)*, a pathogenic gene of Parkinson's disease (PD), has played versatile roles during the disease development. Despite many key insights into LRRK2 studies, the normal and disease-related functions of LRRK2 in microglia and neuroinflammation remain to be fully investigated. Given the importance of LRRK2 in PD pathogenesis, we designed and applied the protocol to target LRRK2. Specifically, we designed two sgRNAs targeting the N terminus of LRRK2, spanning the 5' untranslated region (UTR) and exon 1, and screened knockout cells by single-cell expansion. In practice, the dual-sgRNA system can facilitate in obtaining knockout cells in a more convenient, rapid, and accurate way. Candidate knockout cells can be easily distinguished by genomic PCR and running on agarose gels, based on the different band sizes. Successful knockouts were further verified by Sanger sequencing and Western blot. Using this protocol, we obtained an LRRK2-deficient microglia cell line, which was characterized by longer cellular processes, enhanced adhesion, and weakened migration capacity. The knockout microglia may further serve as an important cellular tool to reveal conserved and novel aspects of LRRK2 functions in the development and progression of PD. Our protocol using dual-sgRNA targeting guarantees > 60% targeting efficiency and could also be applied to targeting other genes/loci, especially non-coding RNAs and regulatory elements.

## Introduction

Parkinson's disease (PD) is the second most common neurodegenerative disease, with clinical manifestations of bradykinesia, muscle rigidity, tremors, and a variety of non-motor symptoms, such as depression, anxiety, or sleep disturbance (de Lau and Breteler, 2006; Hussein et al., 2021). These motor and non-motor symptoms affect at least 1% of people older than 65 years and at least 4% of people older than 80 years, seriously affecting physical/mental health and quality of life of patients (de Lau and Breteler, 2006). Idiopathic PD represents over 90% of PD cases, while hereditary PD represents only 10% of the PD case (Rocha et al., 2022). Despite that, studying the genetic deficiencies in the last few decades has provided us with a clearer etiology of PD. Among them, missense mutations in *leucine-rich repeat kinase 2* (*LRRK2*) are the most common cause of autosomal hereditary PD. Mounting studies have revealed *LRRK2* missense mutations can increase its kinase activity, which causes significant impairments of a variety of cellular physiology, such as autophagy, phagocytosis, and mitochondrial function (Greggio et al., 2006; Zhang et al., 2022).

Numerous studies have demonstrated that the pathogenesis of neurodegenerative diseases has been linked to inflammatory cell activation, impaired cellular and humoral immune responses, autoimmune diseases, and immune dysregulation (Dzamko et al., 2015; Tan et al., 2020; Rostami et al., 2021). Of those, microglia-mediated inflammation has been a shared hallmark of neurodegenerative diseases including PD. Basically, microglia are the resident macrophages of the central nervous system (CNS) and play a key role in maintaining brain homeostasis (Kettenmann et al., 2013; Le et al., 2016). In the normal brain, microglia are considered “resting” and rapidly activated by various types of pathological events or multiple PAMPs/DAMPs (Wolf et al., 2017). Depending on the stimuli, activated microglia can release inflammatory mediators such as tumor necrosis factor-α (TNF-α), interleukin 1 beta (IL-1β), IL-6, reactive oxygen species (ROS), and nitric oxide (NO), as well as neurotrophic factors and repair/clearance factors that would facilitate the phagocytosis of cellular debris and apoptotic cells (Saijo and Glass, 2011). However, the over-activation or persistent activation of microglia may cause a vicious cycle of chronic neural degeneration and pro-inflammation (Tang and Le, 2016; Newcombe et al., 2018; Li et al., 2022). As such, targeting microglia-mediated neuroinflammation might produce promising therapeutic benefits.

Interestingly, LRRK2 is expressed in most immune cells, including microglia, and modulates inflammatory pathways. Dissecting the underlying mechanism of LRRK2-associated neuroinflammation is one of the most common strategies to explore the immunopathogenesis of PD (Russo et al., 2014, 2022). Notably, novel findings on how LRRK2 and its intervention affect the functions of microglia have rapidly emerged (Zhang et al., 2022). For instance, microglia carrying *LRRK*2^G2019S^ exhibited ADP-induced retardation and delayed injury isolation, whereas LRRK2-knockdown microglia are highly motile (Choi et al., 2015). LRRK2 also regulated autophagy in BV2 microglial cells upon LPS treatment, by translocating itself to the autophagosome membrane, whereas loss of LRRK2 resulted in autophagy defects (Schapansky et al., 2014). Microglia from mice with the *LRRK*2^G2019S^ exhibited increased cellular activity and phagocytic responses (Kim et al., 2018; Dwyer et al., 2020). Moreover, the loss, inhibition, or mutation of LRRK2 caused impaired mitochondrial function and degeneration (Cherra et al., 2013; Schwab and Ebert, 2015; Ludtmann et al., 2019).

To study microglial functions, gene deletion in microglia has become an important and routine approach, especially after the development of the CRISPR/Cas9 system for genome editing during the last decade. Current studies on LRRK2 functions largely focused on the gain of function, such as studying the *LRRK*2^G2019S^ mutant. To better investigate the role of LRRK2 in microglia, both loss-of-function and gain-of-function strategies are encouraging to be employed. Previous strategies for loss of function in microglia cells have mostly used small interfering RNAs/short hairpin RNAs (siRNAs/shRNA) or crossing with CX3CR1^CreERT2^ transgenic mice (Wieghofer and Prinz, 2016; Wolf et al., 2020; Arreola et al., 2021). Later on, increasing studies have employed CRISPR/Cas9 by using single short guide (sgRNA) for gene knockout both *in vitro* and *in vivo*, with varied knockout efficiencies (Raas et al., 2019; Raikwar et al., 2019; Wißfeld et al., 2021). In this study, we described a detailed protocol to construct LRRK2-knockout microglia cells *in vitro* by the CRISPR/Cas9 system. Specifically, we designed dual-sgRNA targeting that can facilitate obtaining knockout cells in a more convenient, rapid, and accurate way, compared with the single-sgRNA strategy (Wu et al., 2019). Candidate knockout cells can be easily distinguished by genomic PCR and running on agarose gels, based on the different band sizes. We further used Sanger sequencing and Western blot to verify successful knockouts. Importantly, the obtained LRRK2-deficient microglia were characterized by longer cellular processes, enhanced adhesion, and weakened migration capacity, which is line with previous studies on LRRK2. The knockout microglia can further serve as an important cellular tool to reveal LRRK2 functions during the development and progression of PD. At last, our protocol using dual-sgRNA targeting guaranteed 60% targeting efficiency at a minimum and could be easily applied to targeting other genes/loci.

## Materials and methods

### General supplies

1. Cell culture incubator (HERAcell 150i; Thermo Fisher, Waltham, MA, USA)
2. Trypsin-EDTA (0.25%) (Thermo Fisher, #25200072)
3. 6-Well cell culture plates (Jet Biofil, China, #TCP010006)
4. 24-Well cell culture plates (Jet Biofil, #TCP010024)
5. 96-Well cell culture plates (Jet Biofil, #TCP010096)
6. 3.5-cm cell culture dish (Jet Biofil, #TCD000035)
7. 6-cm cell culture dish (Jet Biofil, #TCD0000660)
8. 10-cm cell culture dish (Jet Biofil, #TCP011001)
9. ChemiDoc XRS^+^ System (Bio-Rad, Hercules, CA, USA, #1708265)
10. T100 PCR Thermal Cycler (Bio-Rad, #1861096)
11. Nano300 Micro-Spectrophotometer (Allsheng, China)
12. BsmBI (NEB, Ipswich, MA, USA, #R0739S)
13. NEBuffer r3.1 (NEB, #B6003V)
14. T4 polynucleotide kinase (T4 PNK) (NEB, #M0201V)
15. 10 x T4 DNA ligase buffer (NEB, #B0202S)
16. T4 DNA ligase (NEB, #M0202V)
17. Polyethyleneimine (PEI; Polysciences, Warrington, PA, USA, #23966)
18. Cryotube vials (Thermo Fisher, #375418PK)
19. Opti-MEM (Thermo Fisher, #31985070)
20. Minimum essential medium (MEM; Procell, China, #PM150410)
21. Dulbecco's modified Eagle medium (DMEM; Procell, #PM150210)
22. Fetal bovine serum (FBS; Transgen Biotech, China, #PS201-02)
23. 100-bp DNA ladder (Tsingke Biotech, China, #TSJ100-100)
24. SanPrep Column Plasmid Miniprep Kit (Sangon Biotech, China, #B518191)
25. Anti-LRRK2 antibody (Abcam, Cambridge, UK, #ab133474)
26. Anti-α-tubulin antibody (Sigma, Darmstadt, Germany, #T5168)
27. Betaine (Sigma, #B2629)
28. EasyTaq DNA polymerase (Transgen Biotech, #AP111-01)
29. Nuclease-free water (Thermo Fisher, #AM9914G)
30. FastAP (Thermo Fisher, #EF0652)
31. TE buffer (Thermo Fisher, #12090015)
32. RIPA buffer (Beyotime, China, #P0013B)
33. Protease inhibitor cocktail (PIC; Transgen Biotech, #DI101-01)
34. 40% acryl/bis (29:1) solution (Sangon Biotech, #B546013)
35. Easy II protein quantitative kit (BCA) (Transgen Biotech, #DQ111-01)
36. Protein phosphatase inhibitor cocktail (PPI; Solarbio, China, #P1260)
37. WesternBright ECL-HRP substrate (Advansta, San Jose, CA, USA, #K-12045-D50)
38. PVDF membrane (Thermo Fisher, #88518)
39. M5 SuperRange protein ladder (10–310 kDa) (Mei5 Biotech, China, #MF290)
40. Antibiotics (penicillin/streptomycin/amphotericin B) (Beyotime, #C0224)
41. Transwell chamber (SPL Life Sciences, Gyeonggi-do, Korea, #35224)
42. 0.45-μm filter (Jet Biofil, #FPE404030)
43. Fluorescence microscope (Leica DMi8, Wetzlar, Germany)

### The strategy of sgRNA design

**Figure 1:**
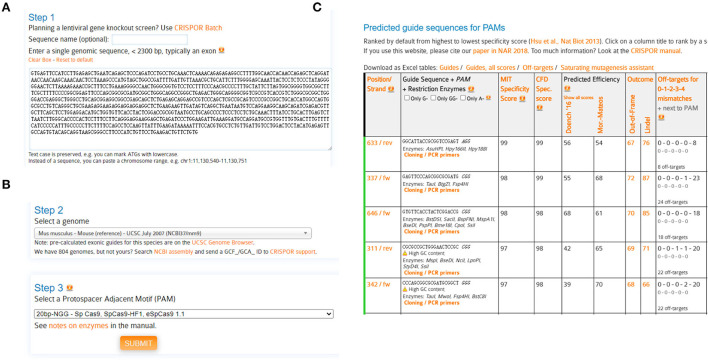
Schematic diagram of sgRNA design. **(A)** Query genomic DNA sequence (containing 5'UTR, exon1, exon2) of LRRK2 with the webserver CRISPOR. **(B)** Select a genome [Mus musculus-Mouse (reference)-UCSC July 2007 (NCBI37/MM9)] and a protospacer adjacent motif (PAM) (20bp-NGG-Sp Cas9, SpCas9-HF1, eSpCas9 1.1). **(C)** Output of predicted guide sequences by CRISPOR. Rank and select sgRNAs with high specificities and efficiencies.

1. We first designed two sgRNAs targeting LRRK2 with the help of CRISPOR website (http://crispor.tefor.net/) (Concordet and Haeussler, 2018).
  **Note:** Other online servers such as CHOPCHOP (http://chopchop.cbu.uib.no) (Labun et al., 2016) and Benchling (https://benchling.com) are also encouraged.
2. The genomic DNA (gDNA) sequence of mouse LRRK2 was retrieved from the NCBI database. Query the N-terminal gDNA sequence (containing LRRK2 5'UTR and exon 1/2) for CRISPOR analysis (Figure 1A).
  **Note:** For gene knockouts, sgRNAs commonly target 5′ constitutively expressed exons, which diminish the chances that the targeted region is removed from mRNA due to alternative splicing. Since genes may have multiple splice isoforms and alternative start sites, it is advisable to target shared coding regions to ensure disruption of all isoforms (Santos et al., 2016). Generally, we would like to introduce insertion/deletion (indel) as close to the 5′ end of the coding region as possible, which will have the highest likelihood of creating a protein-destroying frameshift.
3. Select a genome: Mus musculus-Mouse (reference)-UCSC July 2007 (NCBI37/MM9) (Figure 1B).
  **Note:** Before designing sgRNAs, it is advisable to identify the target genomic sequences, by genomic PCR and Sanger sequencing. The gDNA sequences should be carefully checked to ensure if the target site has any single-nucleotide polymorphism (SNP) that is different from the selected reference genome, which may dampen the binding efficiencies between Cas9 and its DNA target (Lessard et al., 2017; Scott and Zhang, 2017).
4. Select a protospacer adjacent motif (PAM): 20bp-NGG-SpCas9, SpCas9-HF1, eSpCas9 1.1 (Figure 1B), and submit.
5. Selection of candidate sgRNAs.
  Predicted guide sequences were displayed: The #633 and #337 sgRNAs were chosen in this study, due to their highest specificities yet with high cleavage efficiencies (Figure 1C).
  #633 sgLRRK2-1: GAGTTCCCAGCGGCGCGATG
  #337 sgLRRK2-2: GGCATTACCGCGGTCCGAGT
  **Note:** The candidate sgRNAs are chosen based on combined variables, such as cleavage efficiency, specificity, GC content, and sequence constitution. To ensure a successful knockout, the following criteria are basically required:

- The cleavage efficiencies of sgRNAs based on SpCas9 have been evaluated by multiple *in silico* algorithms (Moreno-Mateos et al., 2015; Xu et al., 2015; Doench et al., 2016; Listgarten et al., 2018). The sgRNAs with higher scores have higher possibilities to cut the gDNA.
- For efficient transcription of sgRNA under the U6 promoter, a G is preferred at the 5' position, which corresponds to the first base of the 20-nt sgRNA. For sgRNAs that do not begin with a G, it is recommended to add an additional G, resulting in a 21-nt guide sequence (5'-GN_20_-3') upstream of the PAM site. The addition of a 5' G does not alter the specificity of the sgRNA or affect the efficiency of Cas9 cleavage.
- Never use an sgRNA with >3 Ts in a row since they act as Pol III terminators (for U6 and U3 promoters).
- GC Content: sgRNAs containing intermediate GC content (between ~30 and 80%) outperformed their counterparts with a high or low GC content, in terms of both Cas9 cleavage efficiencies and specificities (Wang et al., 2014; Tsai et al., 2015).
- Specificity and off-target effects.

The specificity score indicates the uniqueness of an sgRNA inside the genome. The higher the specificity score, the lower the potential off-target effects. It is suggested to select the sgRNAs with minimized off-targets. In practice, the possible off-targets could be assessed throughout the genome and rigorously searched by a maximum of 3-nt mismatches, with the help of web servers such as CRISPOR and Cas-Offinder (www.rgenome.net/cas-offinder) (Bae et al., 2014). In this study, sgLRRK2-1 and−2 manifest high specificities (with scores 99 and 98, respectively), except a 3-nt mismatch off-target for sgLRRK2-1 residing at the intergenic region.

**Table:** 

**Off-target seq**	**Mismatch position**	**Mismatch counting**	**Chromosome**	**Start**	**End**	**Strand**	**Locus description**
GAGTTCCaA GCGGCtgGATG	.......*......**....	3	chr19	36324302	36324324	–	Intergenic: Ankrd1-Pcgf5

### Construction of targeting plasmids

1. Targeting plasmids were constructed harboring Cas9 and sgRNA in one plasmid. To this end, sgLRRK2-1 and sgLRRK2-2 sequences were, respectively, cloned into the lentiCRISPR-blasticidin backbone, modified from lentiCRISPR-puro (Addgene #52961).
2. Synthesize two pairs of oligos according to the following form.

sgLRRK2-1:

**Table:** 

Oligo 1	5'	CACCGGAGTTCCCAGCGGCGCGATG	3'
Oligo 2	3'	CCTCAAGGGTCGCCGCGCTACCAAA	5'


sgLRRK2-2:

**Table:** 

Oligo 1	5'	CACCGGGCATTACCGCGGTCCGAGT	3'
Oligo 2	3'	CCCGTAATGGCGCCAGGCTCACAAA	5'


**Note:** Oligos can be synthesized in the standard desalted form or purified by PAGE.

3. Lentiviral vector digestion

The backbone vector (lentiCRISPR-blasticidin) was digested by BsmBI, dephosphorylated, and recovered:

(1) Reaction system:

**Table:** 

**Reagent**	**Volume**
lentiCRISPR-blasticidin (5 μg)	X μl
BsmBI (10,000 U/ml)	3 μl
10 x NEBuffer r3.1	6 μl
DTT (100 mM)	0.6 μl
ddH_2_O	50.4-X μl
	60 μl in total

(2) Digestion was performed at 55 °C for 30–45 min.
(3) Add 1 μl FastAP (1 U/μl) inside the reaction and further incubate at 37°C for 15 min.
(4) Add DNA loading buffer to inactivate BsmBI and AP.
(5) Run on 1% agarose gel and recover the linearized vector (large fragment) from the gel.

**Note:** DTT was freshly prepared and used immediately to avoid decomposition.

2. Anneal and phosphorylate each pair of oligos:

(1) Reaction system:

**Table:** 

**Reagent**	**Volume**
Oligo 1 (100 μM)	1 μl
Oligo 2 (100 μM)	1 μl
10 x T4 DNA Ligase Buffer	1 μl
T4 PNK (10,000 U/ml)	0.5 μl
ddH_2_O	6.5 μl
	10 μl in total

(2) Phosphorylation/annealing reaction was placed in a thermocycler with the following parameters:
  37°C 30 min
  95°C 5 min
  Ramp down to 25°C at 5°C/min (0.1°C/s).

(3) Annealed oligos were diluted at 1:200 in sterile water or elution buffer (Sangon Biotech).
  **Note:** The T4 DNA ligase buffer was used since the buffer supplied with the T4 PNK enzyme does not include ATP. Otherwise, 1 mM ATP should be added.

3. Ligation and transformation:

(1) Reaction system:

**Table:** 

**Reagent**	**Volume**
Recovered BsmBI digested plasmid (50 ng)	X μl
Diluted oligos duplex	1 μl
10 x T4 DNA Ligase Buffer	1 μl
T4 DNA Ligase (400,000 U/ml)	0.5 μl
ddH_2_O	7.5-X μl
	10 μl in total

(2) The reaction was allowed to incubate for 30 min at room temperature (RT).
(3) Transform ligated products into competent bacteria.

**Note:** Since lentiviral plasmids basically contain long-terminal repeats (LTRs), the transformation should be performed in recombination-deficient bacteria, such as Stbl3 bacteria.

### Cell culture

BV2 microglial cells (ATCC, #CRL-2467) were cultured in MEM supplemented with 10% FBS and 1% antibiotics. 293T cells (ATCC, #CRL-3216) were cultured in DMEM supplemented with 10% FBS and 1% antibiotics. All cells were routinely cultured in a humidified incubator in which 5% CO_2_ was supplied and maintained at 37°C.

**Note:** FBS used for culturing BV2 cells should be inactivated at 56°C for 30 min.

### Transfection and lentivirus package

Considering that microglia are difficult cells to transfect, lentivirus package was thus employed.

1. Lentiviral and helper plasmids were expanded in LB medium and extracted using a plasmid miniprep kit.

**Note:** The plasmids over 1 μg/μl may increase the efficiency of the virus package. As such, it would be better to prepare plasmids by midi- or maxipreps.

2. At 1 day before transfection, 293T cells were digested with 0.1% trypsin, followed by seeding on 10-cm dishes at a density of 3 × 10^6^ cells/dish.
3. Transfections were performed upon approximately 60%~80% confluency. The following is the transfection system:

**Table:** 

**Reagent**	**Volume**
Opti-MEM	1 ml
psPAX2	4.5 μg
PMD2.G	3 μg
LentiCRISPR-sgLRRK2-1 or−2	6 μg
PEI	40.5 μl

4. First, all plasmids were diluted with 1 ml Opti-MEM, respectively, in 1.5-ml EP tubes, followed by adding desired amounts of PEI. The tubes were then mixed gently and stood at RT for 15 min. The mixtures were finally added to 10-cm dishes.

**Note:** The amount of PEI (μl) is 2–3 times the amount (μg) of all plasmids combined.

5. After overnight, the cells were washed with DPBS and replaced with fresh medium.

**Note:** Caution should be taken while washing cells and changing fresh medium since 293T cells can easily float.

6. Culture medium containing lentiviruses were, respectively, collected at 48 and 72 h post-transfection and were filtered through 0.45-μm filters before use.

**Note:** The virus supernatant can be used directly as soon as possible or concentrated. The remaining viruses can be stored at −80°C for further use, albeit with reduced titer.

### Lentiviral infection of BV2 cells

1. For infection, BV2 cells were seeded into 3.5-cm dishes at a density of 3 × 10^5^ cells/dish. At the same time, each dish was added with 3 ml of lentivirus supernatant (MOI = 100) and polybrene (8 μg/ml).
2. On the next day, the culture was replaced with a fresh medium and observed daily.
3. On day 3, BV2 cells were seeded into 6-cm dishes and treated with blasticidin (10 μg/ml). Part of the survived cells can be frozen as stock before single clone selection.

### Single clone selection

1. To ensure single-cell growth, the survived BV2 cells were diluted to a concentration of 5 cells/ml and seeded into a 96-well plate.
  **Note:** In our protocol, seeding 0.5 cells/100 μl per well in a 96-well plate can reduce the possibility of existence of multiple colonies. However, the desired dilution will need to be adapted depending on the specific situation.
2. The cell growth were routinely observed under the microscope.
  **Note:** At early stages, the cell colonies in each well should be carefully checked to make sure that they grow from single cells. The candidate cells were marked in advance.
3. Upon confluency, half of candidate cells were passaged to a 48-well plate for continuous growth; another half were spin down and harvested for gDNA extraction and genotyping by junction PCR.
  **Note:** For single cell selection, we also practiced manual picking by a pipette. Specifically, BV2 cells were seeded on a 10-cm dish (5,000 cells) and grew for picking. However, it is quite tricky for manual picking since BV2 cells are small in size and tightly adhere to the dish. As such, BV2 cells are easily squeezed and deformed by the pipette tip. Thus, manual picking is not recommended.

### Genomic DNA isolation

1. Cell pellets were digested with DNA lysis buffer containing proteinase K (0.5 mg/ml) overnight at 55°C in a water bath.
2. On the next day, equal volumes of phenol:chloroform:isoamyl alcohol (25:24:1) were added into EP tubes, followed by vortexing and spinning at 14,000 rpm for 15 min.
3. The upper phase was carefully transfer into new EP tubes, and 1/10 volume of 3 M sodium acetate (pH5.2) and 2–2.5 volume of 100% ethanol were added.
4. The tubes were placed at −20°C (or −80°C) for >30 min until DNA precipitate forms.
5. The extracts were subsequently separated by centrifugation at 14,000 rpm for 10 min at 4°C. The supernatant was discarded.
6. DNA pellets were then washed with 1 ml of 70% ethanol and centrifuged at 14,000 rpm for 5 min.
7. The supernatant were carefully removed, and the DNA pellets were allowed to air dry.
8. A measure of 50 μl TE buffer was added into the EP tubes, and they were incubated at 37°C for 10 min to resuspend gDNA.
9. DNA concentrations were measured with the spectrophotometer and were later diluted to a final concentration of 150 ng/μl with ddH_2_O.

### Junction PCR

The candidate knockouts could be easily distinguished by junction PCR and running on agarose gels, based on different band sizes.

**Note:** The dual-sgRNA targeting is possible to delete a length of gDNA between sgRNAs. This provides a unique advantage for visualizing edited events without sequencing.

**Figure 2:**
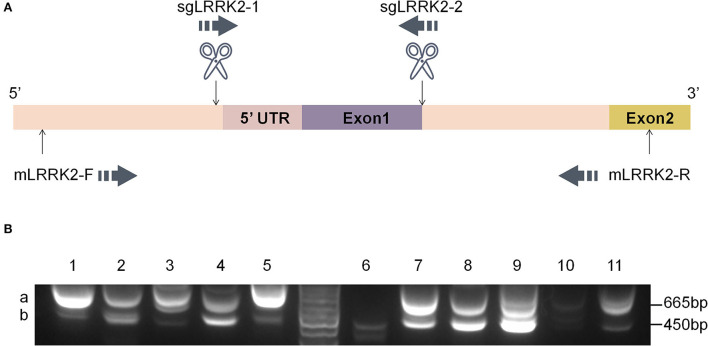
Dual-sgRNA targeting strategy. **(A)** A pair of sgRNAs were designed to target the N terminus of *LRRK2*; sgLRRK2-1 is located upstream of the 5'UTR, and sgLRRK2-2 spans exon 1/intron 1. The primers for genomic PCR, mLRRK2-F and mLRRK2-R, were located upstream and downstream of the two sgLRRK2s, respectively. **(B)** Candidate LRRK2-KO cells were expanded from single cells (#1–#11) and were examined by junction PCR on gDNAs.

1. Sequences of PCR primers are listed as follows (Figure 2A):

**Table:** 

**Junction PCR**	**Sequences (5^′^-3^′^)**
mLRRK2-F	CCATGTAGCTGGCCGATTTTG
mLRRK2-R	GGACAATCAACAGAGGCACGT

2. PCR system:

**Table:** 

**Reagent**	**Volume**
gDNA Template (150 ng/μl)	1 μl
mLRRK2-F (10 μM)	0.4 μl
mLRRK2-R (10 μM)	0.4 μl
10 x EasyTaq Buffer	2 μl
dNTP (2.5 mM)	1.6 μl
Easy Taq DNA Polymerase (5 U/μl)	0.4 μl
4 x Betaine (4 M)	5 μl
ddH_2_O	X μl
	20 μl in total

3. PCR was placed in a thermocycler with the following parameters:

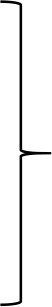

**Figure 3:**
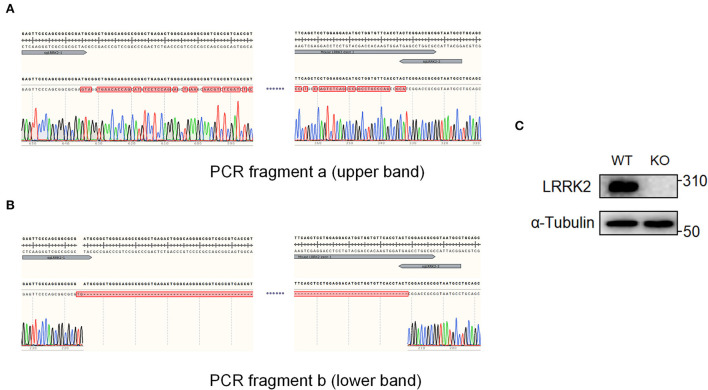
Candidate knockout clones were validated by Sanger sequencing and Western blot. The two PCR fragments, a and b of single clone #7 in Figure 2B, were further sequenced, respectively. **(A)** Sequencing result revealed extensive nucleic changes between two sgRNAs, which has caused a frameshift of LRRK2 ORF and finally led to premature stop of translation. **(B)** Sequencing of PCR fragment b showed that the genomic region, harboring 5'UTR and exon 1 of LRRK2 between two sgRNAs, has been successfully cleaved. **(C)** LRRK2 protein expression was verified by Western blot.

4. PCR products were run in 1% agarose gel.
5. The sizes of PCR bands were observed to identify candidate knockouts (Figure 2B).
6. Gels containing the desired bands were cut out for further Sanger sequencing (Figures 3A,B).

**Note:** Since each clone might be derived from several single clones, Sanger sequencing is required to verify the knockout genotypes. In case of multiple sequencing reads from one PCR band, the selected cells are highly possible mixed, which will need to be diluted for a second round (step 2.7) to finally screen out the single ones.

### Protein isolation and western blot

The candidate knockouts were further verified by Western blot to make sure that the coding region of target gene has been successfully disrupted.

1. RIPA buffer supplemented with 1% PIC, 1% PPI, and 1% EDTA were pre-prepared.
2. BV2 cells grown in six-well plates were washed two times with ice-cold PBS.
3. A measure of 100 μl RIPA buffer was added per well of six-well cell culture plates, and cells were harvested by scraping into EP tubes.
4. Cells were lysed by ultrasonication and placed on ice for 30 min.
5. Cell lysates were then centrifuged at 12,000 rpm for 30 min at 4°C.
6. The supernatant was decanted into new 1.5-ml EP tubes. The isolated proteins were stored at −80°C.
7. Protein concentrations were determined by using the BCA method, according to the user manual.
8. A measure of 20 μg of proteins were taken out from each sample and were added with 4 x SDS loading buffer, followed by boiling at 98°C for 10 min.
9. To perform SDS-PAGE, an equal amount of protein samples was loaded onto an 8% polyacrylamide gel.
10. The electrophoresis apparatus was filled with 1 x SDS running buffer. The gel was run at 80 V for 30 min, followed by 170 V for 1–2 h.
11. Then, 0.45-μm PVDF membranes were prepared by wetting the gel with methanol for 30 s.
12. To transfer proteins onto the membranes, a ‘sandwich' containing gel, membrane, and filter papers was built, which moved toward the positive electrode.
13. 1 x Transfer buffer was added to the electrophoresis tank, and the transfer was started at 300 mA for 90 min.
14. The membranes were then taken out and blocked with 5% skim milk for 1 h at RT.
15. The primary antibodies was diluted to working concentration in 5% skim milk. The membranes were incubated with primary antibodies for 16–18 h at 4°C.
16. On the next day, membranes were washed three times with TBST buffer at a 15-min interval.
17. Secondary HRP-conjugated antibodies were diluted to working concentration with 5% skim milk and were used to incubate with membranes for 1 h at RT.
18. The membrane was washed again with TBST buffer three times at a 15-min interval.
19. The HRP substrates were then freshly prepared and added onto membranes. The luminescence was finally visualized using a Chemiluminescence Imager (Figure 3C).

### Migration assay

1. BV2 microglial cells were digested with 0.25% trypsin, which was quenched by adding an equal volume of MEM containing 10% FBS. The cells were centrifuged at 800 rpm for 4 min, and the supernatant was discarded.
2. The cell pellets were suspended with 1% FBS medium at a density of 3 × 10^5^ cells/ml.
  **Note:** Cell migration is initiated using different FBS concentrations between the medium inside or outside of the chamber.
3. Transwell chambers were placed into a 24-well plate. Then, 100 μl of suspended cells were seeded into the Transwell chamber, followed by adding 600 μl of MEM containing 10% FBS outside the chamber of each well.
4. The transmigration experiment was started. The cells were incubated in the 24-well plate inserts in an incubator (37°C, 5% CO_2_) for 24 h.
5. The chamber was removed, placed into a beaker, and rinsed with PBS three times.
  **Note:** Since cells are easily dropped off from the chamber, the chamber should be gently handled before fixation.
6. The cells were fixed by adding 500 μl of 4% paraformaldehyde to each well for 2 min.
7. The chamber was removed, placed into a beaker, and rinsed with PBS three times.
8. A measure of 500 μl of methanol was added to each well and incubated at RT for 20 min.
9. The chamber was removed, placed into a beaker, and rinsed with PBS three times.
10. Then, 500 μl of 0.1% crystal violet staining solution was added to each well and incubated at RT for 20 min.
11. After that, the chamber was removed, placed into a beaker, and rinsed with PBS two times.
12. Non-migrated cells on the upper side of the membrane was removed using a cotton swab.
13. **Note:** The cotton swab must be cautiously used to avoid wiping the migrated cells off of the lower side of the membrane.
14. The migrated cells were observed under the microscope, and five visual fields were randomly chosen for taking pictures and further data processing.
  **Note:** To visualize more significant differences, consider culturing the cells with serum-free medium for 12–24 h before starting the migration assay.

### Statistical analysis

All graphs were performed by GraphPad Prism version 9 software. Student's *t*-test was applied at 95% confidence interval to determine the statistical differences between groups, with p < 0.05 indicating statistical significance.

## Results and discussion

### Generation of LRRK2-knockout microglia cells using the CRISPR/Cas9 system

We applied dual-sgRNA targeting by the CRIPSR/Cas9 system aiming to generate knockout cells in a more convenient and rapid way. Specifically, two sgRNAs targeting the N-terminal LRRK2 (sgLRRK2-1 and sgRRK2-2, respectively) were designed to disrupt its expression (Figure 2A). sgLRRK2-1 is located before the 5' UTR region, and sgLRRK2-2 spans exon 1/intron 1. As shown in Figure 2A, both sgLRRK2-1 and sgLRRK2-2 targeting may efficiently cleave the gDNA and cause a gDNA deletion of 299 bp in length.

After the lentiviral infection of Cas9 and sgRNAs, BV2 microglial cells were screened by blasticidin, and survived cells were further expanded as single cells. We then selected 11 single clones and performed genotyping by junction PCR on gDNAs. Excitingly, we observed that seven of 11 clones had two PCR bands (Figure 2B; Supplementary Figure 1), indicating that the editing event of gDNA deletion has occurred with a high efficiency (>60%). We further selected #7 clone as a knockout candidate and harvested the upper band “a” and the lower band “b” for Sanger sequencing (Figure 2B).

The sequencing results showed that the region between sgRNAs in undeleted allele (band “a”) has been effectively edited and extensively repaired by non-homologous end joining (NHEJ), leading to premature stop codons within the open reading frame (ORF) of LRRK2 (Figure 3A). In the other allele (band “b”), a length of gDNA including 5'UTR and exon 1 has been successfully deleted. The deletion appears not to be seamless, but instead causes a 2-bp insertion in between sgRNAs (Figure 3B). It is highly possible that both sgRNAs led to 1-bp insertion, followed by the genomic repair, since the most common form of indels favors 1-bp insertion (Allen et al., 2018). In addition, we verified the LRRK2 knockout by Western blot (Figure 3C). Therefore, the #7 clone is proved to be genotypic heterozygous and have completely lost LRRK2 expression.

### Enhanced adhesion and altered morphology in LRRK2-KO microglia

**Figure 4:**
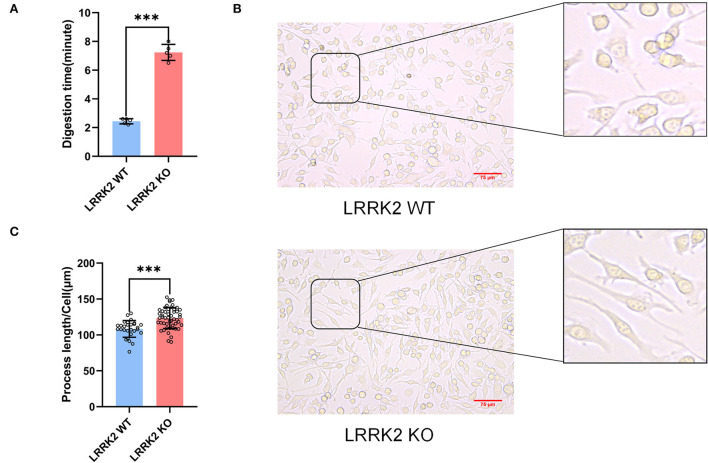
Enhanced adhesion and lengthened process of microglia after LRRK2 knockout. **(A)** Statistics of the time required for trypsinization of LRRK2-WT microglia and LRRK2-KO microglia (*n* = 5 experimental replicates, ***p < 0.001). **(B)** LRRK2-KO microglia showed significantly lengthened process (*n* = 50 fields of view) when compared to WT (*n* = 28 fields of view). Scale bars, 75 μm. **(C)** Statistical analysis of the lengths of cell processes in **(B)** (***p < 0.001).

Previous studies have demonstrated that alterations in microglial morphology and activities are strongly linked to neuroinflammation. During the passage of LRRK2-WT and LRRK2-KO BV2 microglial cells, we found that LRRK2-KO microglia adhered more tightly to the culture dish. Basically, the digestion time for KO cells was around 7 min, whereas the digestion time for WT microglia is only about 3 min (Figure 4A). Moreover, we observed that the cell process of microglia was significantly prolonged after LRRK2 knockout (Figure 4B). This is in line with a previous study which showed that upon LRRK2 knockdown, microglia was morphological polarized and firmly attached to the substratum, whereas WT cells attached weakly to the dish and displayed shorter processes with relatively round shapes (Choi et al., 2015).

### Migration deficits in LRRK2-KO microglia

**Figure 5:**
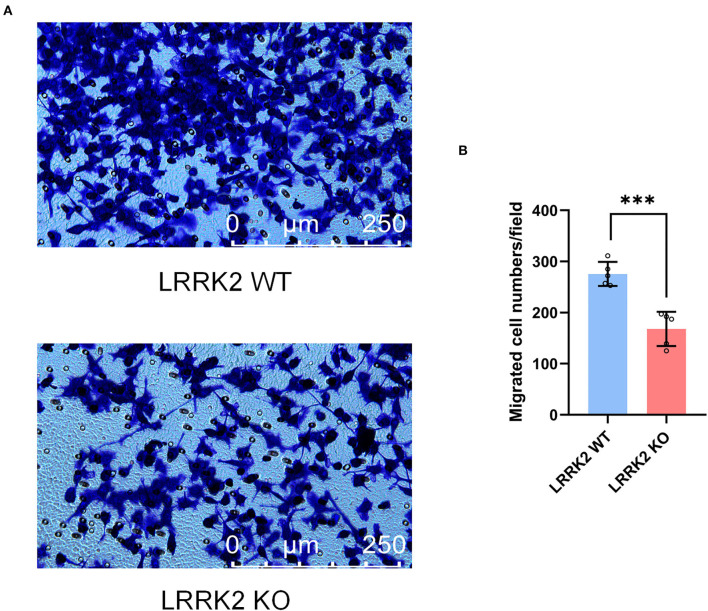
Microglial motility is retarded by LRRK2 knockout. **(A)** Effects on microglial migration after LRRK2 knockout, as assessed by the Transwell migration assay. **(B)** Statistical analysis of migrated cell numbers in **(A)** (*n* = 5 experimental replicates, ***p < 0.001).

Microglia, resident immune cells of the CNS, are capable of migrating toward injury sites and keeping motile to maintain surveillance of brain homeostasis. To further examine the migration ability upon LRRK2 knockout, we performed Transwell assay to assess the migration of both LRRK2-WT and LRRK2-KO microglia. We showed that the number of LRRK2-KO migrated microglia in the lower layer of Transwell chamber was significantly less than that of LRRK2-WT microglia (Figure 5). To be consistent, microglia were less responsive to brain injury in *LRRK*2^G2019S^ transgenic mice, delaying the sequestration of the injury site relative to the surrounding normal tissue (Choi et al., 2015). Thus, our data support that LRRK2 may be required to mediate microglial motility that responds to multiple types of PAMPs/DAMPs.

## Strengths and pitfalls

Our protocol using dual-sgRNA targeting guarantees 60% targeting efficiency at a minimum and could be easily applied to targeting other genes/loci in microglia. By distinguishing the deleted genotypes based on gDNA PCR, the whole procedure could be greatly accelerated, and it basically takes < 1 month. The dual-sgRNA strategy is particularly important for targeting non-coding RNAs or other regulatory elements, such as superenhancers, the gDNA of which will have to be entirely or largely deleted due to their untranslated nature. It should be noted that in recent years, long non-coding RNAs such as Lnc-Gas5, Lnc-MALAT1, and Lnc-Nostrill have been demonstrated to be important regulators during microglial activation and neuroinflammation (Sun et al., 2017; Cai et al., 2020; Mathy et al., 2021). Our protocol thus holds a unique and promising advantage for studying the mechanisms of non-coding RNAs and regulatory elements in modulating neuroinflammation.

Our protocol employed lentiviruses to deliver Cas9 and sgRNAs since microglial cells are generally difficult to be transfected using the traditional liposomes and are quite vulnerable to death. However, the integrated nature by lentivirus might introduce extra risks of unpredictable genomic changes. Alternatively, the nucleofection of plasmids or ribonucleoprotein (RNP) complex consisting of Cas9 protein and sgRNAs might be applied in our protocol. The usage of RNPs has emerged as a critical method due to their advantages of transient genome editing and reduced off-targets.

To comprehensively assess the knockout cells, off-target effects is an important variable to be considered. Basically, the off-target sites, predicted by *in silico* algorithms, can be amplified by genomic PCR for further Sanger sequencing. Note that off-targets located within coding regions/exons may bring grave harms and should be avoided. To examine other potential genomic alterations, knockout cells can be thoroughly and rigorously assessed by deep sequencing or whole-genome sequencing (WGS).

## Data availability statement

The original contributions presented in the study are included in the article/Supplementary material, further inquiries can be directed to the corresponding author.

## Author contributions

YT conceived and designed the study. MZ and YT prepared the draft and figures. All authors have read, revised, and agreed to the published version of the manuscript.

## Funding

This study was funded by the National Key R&D Program of China (No. 2022ZD0213700), National Natural Sciences Foundation of China (Nos. 81801200 and 82271280 to YT; 81901223 to FY), Hunan Provincial Natural Science Foundation of China (No. 2019JJ40476 to YT; 2022JJ40824 to JW), Talents Startup Fund (No. 2209090550 to YT), and Youth Science Foundation (No. 2021Q04 to JW) of Xiangya Hospital, Central South University, Changsha, China.

## Conflict of interest

The authors declare that the research was conducted in the absence of any commercial or financial relationships that could be construed as a potential conflict of interest.

## Publisher's note

All claims expressed in this article are solely those of the authors and do not necessarily represent those of their affiliated organizations, or those of the publisher, the editors and the reviewers. Any product that may be evaluated in this article, or claim that may be made by its manufacturer, is not guaranteed or endorsed by the publisher.

## Supplementary material

Supplementary Figure 1 Candidate LRRK2-KO cells were expanded from single cells and were examined by junction PCR on gDNAs.

Supplementary Figure 2 LRRK2 protein expression was verified by Western blot.

Supplementary Figure 3 Tubulin protein expression was verified by Western blot.

Supplementary Figure 4 LRRK2-WT and LRRK2-KO cells were examined by junction PCR on gDNAs.
