# Supplementary figures and images for: The efficient generation of knockout microglia cells using a dual-sgRNA strategy by CRISPR/Cas9
Source: Front Mol Neurosci. 2022 Oct 13;15:1008827. doi: 10.3389/fnmol.2022.1008827 (PMC9614382; doi:10.3389/fnmol.2022.1008827)

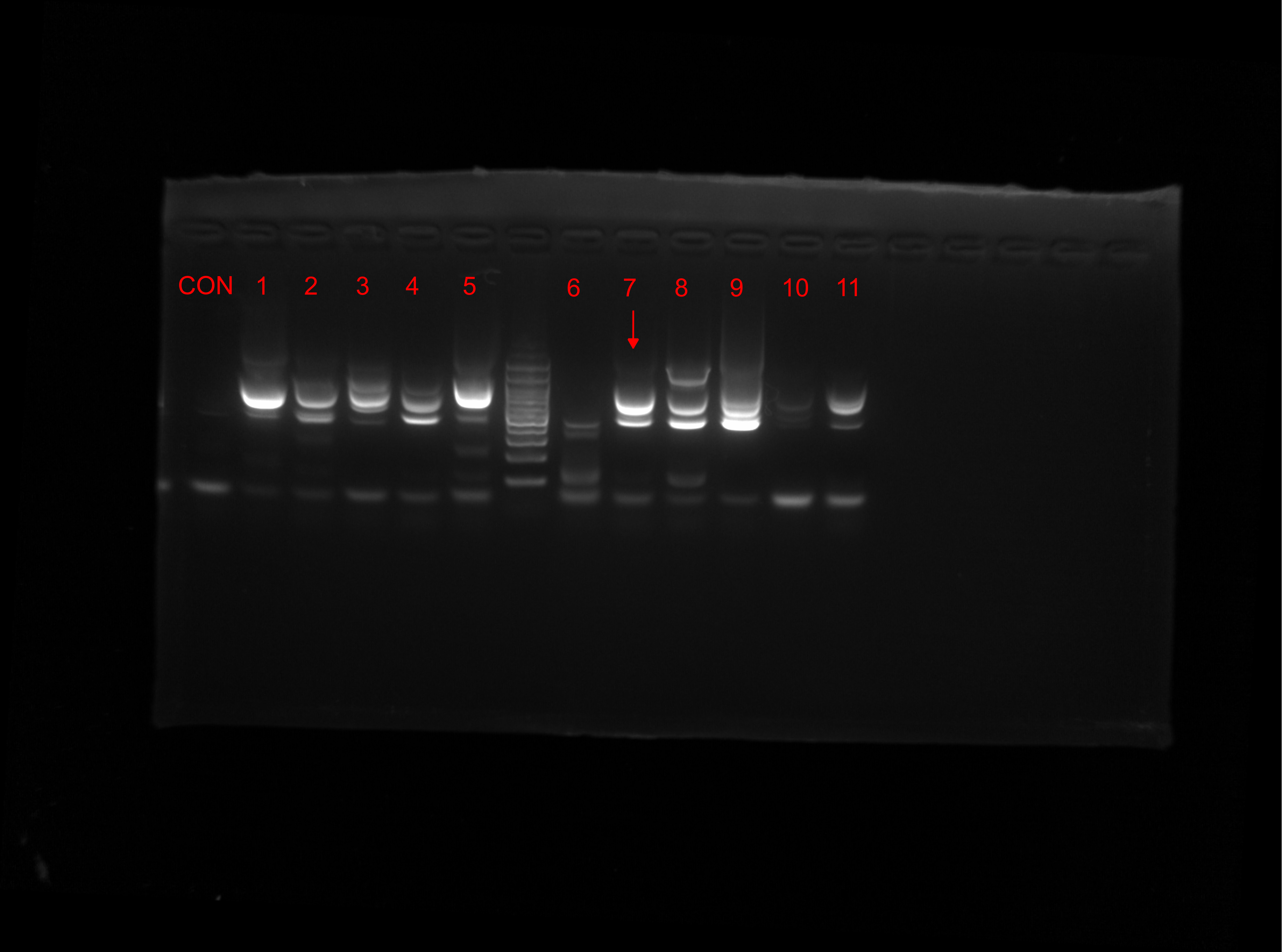

Supplement: Supplementary Figure 1 — Candidate LRRK2-KO cells were expanded from single cells and were examined by junction PCR on gDNAs. [file Image_1.TIF]

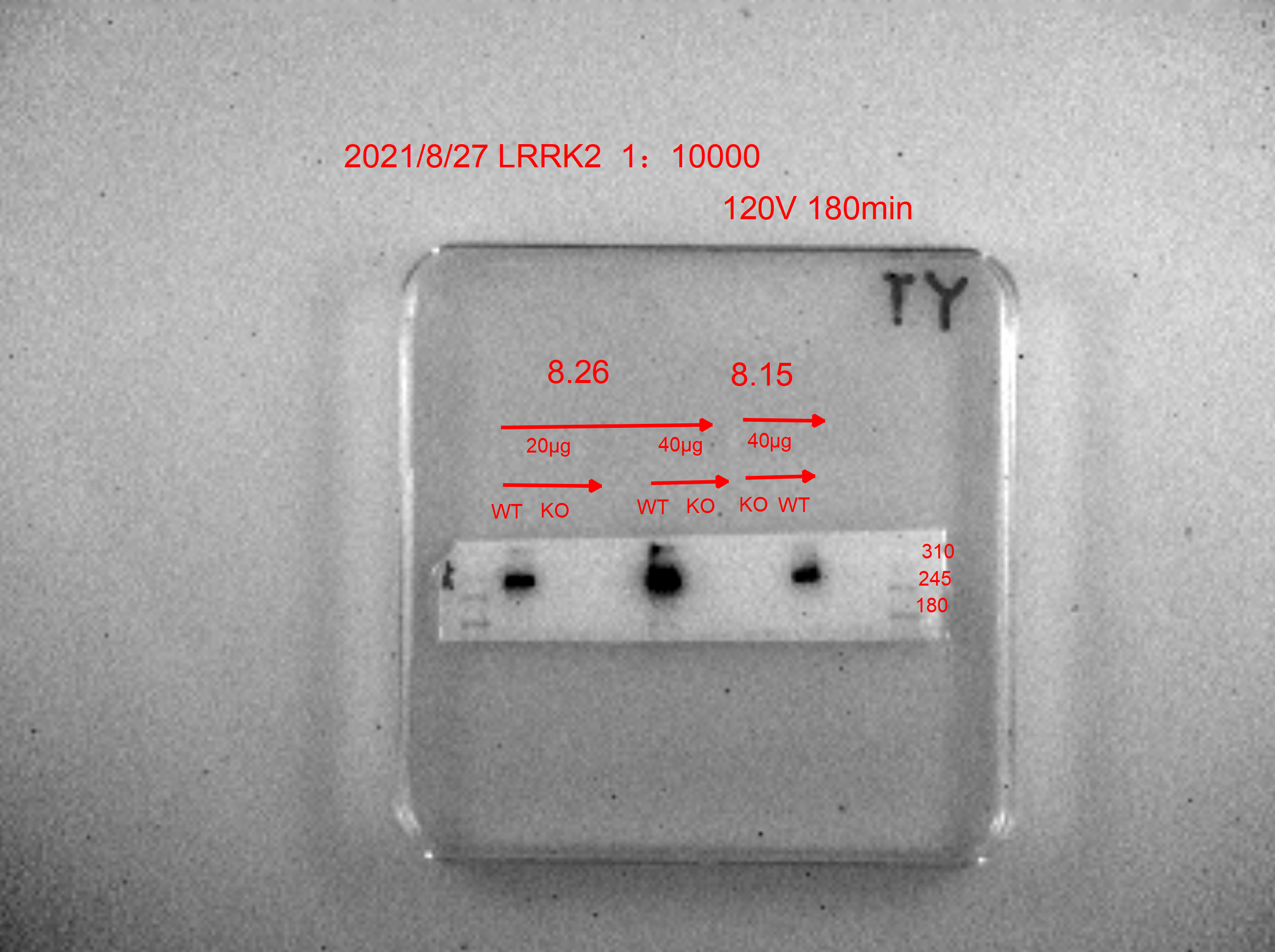

Supplement: Supplementary Figure 2 — LRRK2 protein expression was verified by Western blot. [file Image_2.TIF]

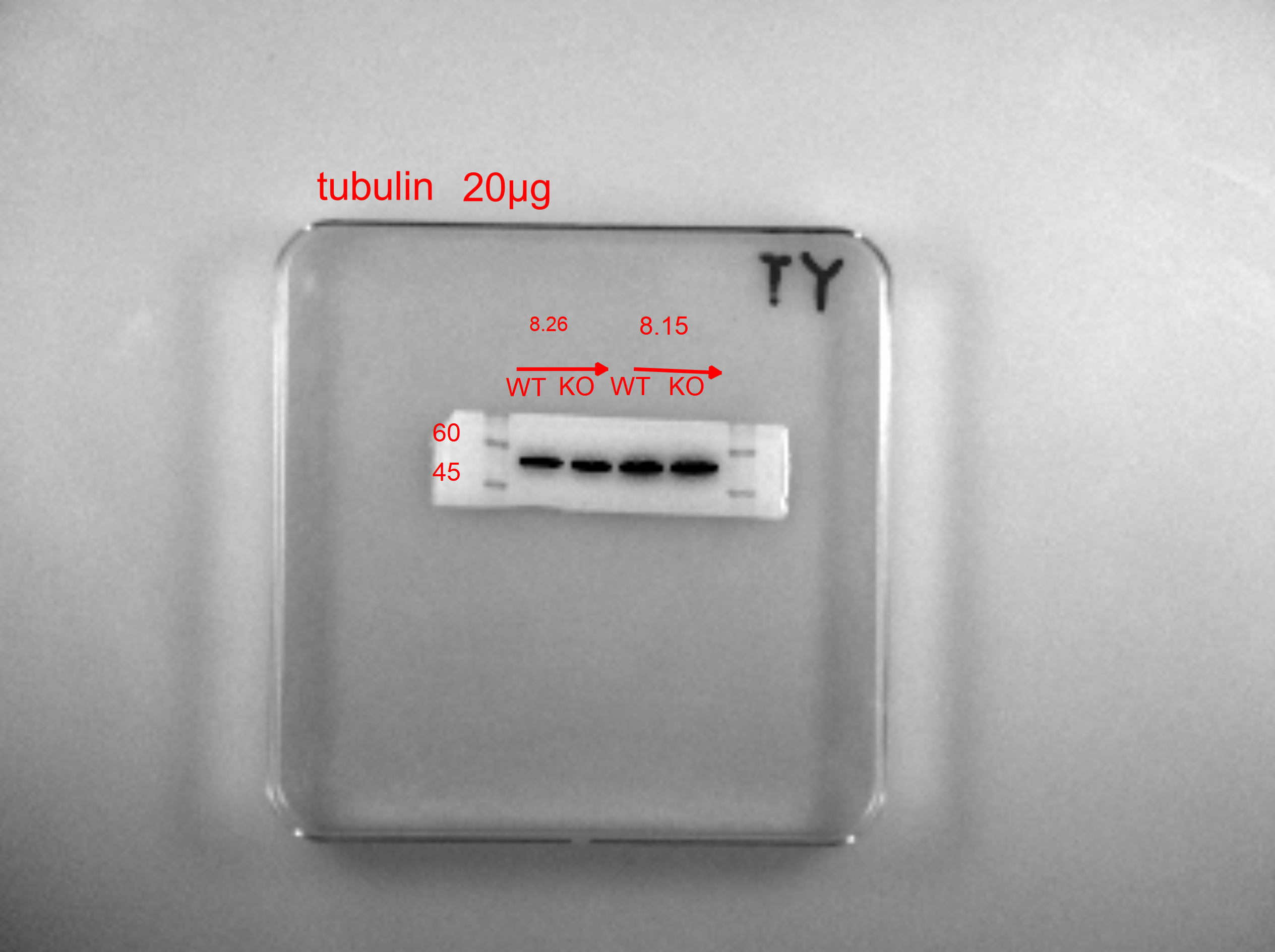

Supplement: Supplementary Figure 3 — Tubulin protein expression was verified by Western blot. [file Image_3.TIF]

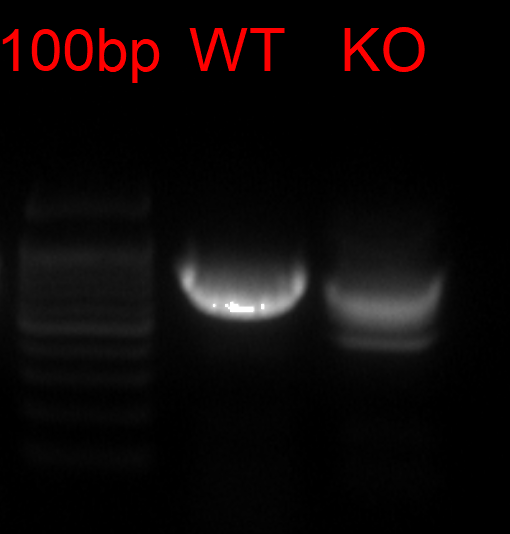

Supplement: Supplementary Figure 4 — LRRK2-WT and LRRK2-KO cells were examined by junction PCR on gDNAs. [file Image_4.TIF]
